# Miscarriage rates after dehydroepiandrosterone (DHEA) supplementation in women with diminished ovarian reserve: a case control study

**DOI:** 10.1186/1477-7827-7-108

**Published:** 2009-10-07

**Authors:** Norbert Gleicher, Eddy Ryan, Andrea Weghofer, Sonia Blanco-Mejia, David H Barad

**Affiliations:** 1 The Center for Human Reproduction-New York and the Foundation for Reproductive Medicine, New York, NY, USA; 2 Department of Obstetrics, Gynecology and Reproductive Sciences, Yale University School of Medicine, New Haven, CT, USA; 3 Toronto West Fertility Associates, Toronto, Canada; 4 Department of Obstetrics and Gynecology, Vienna University School of Medicine, Vienna, Austria; 5 Departments of Epidemiology and Social Medicine and Obstetrics, Gynecology and Women's Health, Albert Einstein College of Medicine, Bronx, NY, USA

## Abstract

**Background:**

Dehydroepinadrosterone (DHEA) supplementation improves pregnancy chances in women with diminished ovarian reserve (DOR), by possibly reducing aneuploidy. Since a large majority of spontaneous miscarriages are associated with aneuploidy, one can speculate that DHEA supplementation may also reduce miscarriage rates.

**Methods:**

We retroactively compared, utilizing two independent statistical models, miscarriage rates in 73 DHEA supplemented pregnancies at two independent North American infertility centers, age-stratified, to miscarriages reported in a national U.S. in vitro fertilization (IVF) data base.

**Results:**

After DHEA supplementation the miscarriage rate at both centers was 15.1% (15.0% and 15.2%, respectively). For DHEA supplementation Mantel-Hänszel common odds ratio (and 95% confidence interval), stratified by age, was significantly lower, relative to odds of miscarriage in the general IVF control population [0.49 (0.25-0.94; p = 0.04)]. Miscarriage rates after DHEA were significantly lower at all ages but most pronounced above age 35 years.

**Discussion:**

Since DOR patients in the literature are reported to experience significantly higher miscarriage rates than average IVF patients, the here observed reduction in miscarriages after DHEA supplementation exceeds, however, all expectations. Miscarriage rates after DHEA not only were lower than in an average national IVF population but were comparable to rates reported in normally fertile populations. Low miscarriage rates, comparable to those of normal fertile women, are statistically impossible to achieve in DOR patients without assumption of a DHEA effect on embryo ploidy. Beyond further investigations in infertile populations, these data, therefore, also suggest the investigations of pre-conception DHEA supplementation in normal fertile populations above age 35 years.

## Background

Casson et al. were first to suggest that dehydroepiandrosterone (DHEA) supplementation may improve selected aspects of ovarian function in women with diminished ovarian reserve [1]. Because they reported only rather small benefits from a short term supplementation protocol, their observation failed to attract follow up. This, however, changed when a woman of advanced reproductive age, after self medication with DHEA, experienced surprising gains in ovarian function [2]. That experience led to a series of studies, investigating DHEA supplementation in infertile women with significant degrees of diminished ovarian reserve.

Those studies suggested that DHEA supplementation improves response to ovarian stimulation with gonadotropins by increasing oocyte yield and embryo numbers [2,3]. Explaining the rather small benefits initially observed by Casson et al after only short-term use [1], DHEA effects increase over time, reaching peaks after approximately four to five months of supplementation [2,4]. DHEA, however, also increases oocyte and embryo quality [3,4], spontaneous pregnancy rates in prognostically otherwise highly unfavorable patients on no further active treatments [4], pregnancy rates with in vitro fertilization (IVF) [4], time to pregnancy and cumulative pregnancy rates [4].

Why DHEA would positively affect ovarian function parameters and pregnancy chances in women with diminished ovarian reserve is still unknown. Casson and colleagues suggested that the effect may be insulin-like growth factors (IGF-1) - mediated [5]. Because DHEA effects peak at four to five months, a time period similar to the complete follicular recruitment cycle, we have speculated about a DHEA effect on follicular recruitment, possibly mediated via suppressive effects on apoptosis [3,4]. Following a small pilot study, of insufficient statistical power, we also noted the possibility that DHEA may reduce aneuploidy in embryos [6].

Since approximately 80 percent of spontaneous pregnancy loss is the consequence of chromosomal abnormalities [7], reduced aneuploidy should also reduce miscarriage rates. As women get older, and ovarian function progressively declines, miscarriage rates rise because of increasing aneuploidy [8,9]. If DHEA, indeed, were to beneficially affect ploidy, DHEA supplementation should, as an additional benefit in older women with severely diminished ovarian reserve, therefore, result in reduced miscarriage rates.

Since women with diminished ovarian reserve produce only small oocyte and embryo numbers with IVF [6,9], preimplantation genetic diagnosis (PGD) in association with IVF is only rarely indicated, and, indeed, may be detrimental [10]. To accumulate direct embryo ploidy data in such patients is, therefore, difficult. Seeking alternatives, we were attracted by the fact that spontaneous miscarriage rates to such a large degree reflect aneuploidy rates. This study, therefore, presents pregnancy outcomes after DHEA supplementation from two independent North American fertility centers and compares those with age-specific national USA outcome data after IVF.

## Methods

### DHEA supplementation

After approval by the center's Institutional Review Board, the Center for Human Reproduction (CHR) in New York City has been utilizing DHEA supplementation in women with diminished ovarian reserve since 2004. Based on reported clinical experiences [1-4], the indications for such supplementation have changed over the years: In initial stages, only older women, above age 42, were supplemented and only if they had failed at least one IVF cycle and less than 4 oocytes had been retrieved in confirmation of ovarian resistance to stimulation. By mid-2005, indications were expanded to all women above age 40 with evidence of ovarian resistance and a history of one failed prior IVF cycle. By early 2006 indications were further expanded to women under age 40 if they demonstrated elevated baseline follicle stimulating hormone (FSH) levels above 10 mIU/ml and had shown ovarian resistance in at least one failed IVF cycle. By mid-2006 FSH baseline criteria were changed from absolute FSH elevations to elevations in age-specific FSH levels [11]. All women above age 40 have been offered routine supplementation since January 2007, while younger women, under age 40, are continuing to be only selectively supplemented if demonstrating elevated age-specific baseline follicle stimulating hormone (FSH) levels and, as previously reported, inappropriately low oocyte yield in at least one IVF cycle [11].

DHEA supplementation in all patients involves oral, pharmaceutical grade micronized medication at a dosage of 25 mg, three times daily (TID). Only morbidly obese women receive an increased daily dosage of 100 mg and no such women were involved in this study. This supplementation dosage was chosen since it represented the amount of medication the index patient used [2] after reading the report by Casson et al. [1]. Limited patient volume and funding sources have prevented dose response studies and 25 mg DHEA TID daily has, therefore, remained the only standard treatment dosage. Patients receive at least two months of DHEA supplementation prior to oocyte retrieval, unless they conceive spontaneously during that time period [4]. This minimum pretreatment period is based on the recognition that at two months pregnancy curves between DHEA pretreated and control patients statistically diverge [4]. DHEA is maintained until pregnancy, and is discontinued with second positive pregnancy test.

### Collaboration between centers

The utilization of DHEA at the Toronto-based center was independently initiated, after that center's medical director (E.R.) at a lecture (by N.G.) learned about the New York center's DHEA experience. Toronto's data accumulation was unknown to the New York center until in December of 2007, unsolicited, a detailed electronic record of Toronto's DHEA experience was forwarded to New York with a request for combined analysis. The Canadian data were sequestered to the New York center's confidential research data base, which is restricted to one computer. Confidentiality and anonymity of submitted records was, therefore, maintained.

### Control population

This study reports on miscarriage rates, at both fertility centers, independently established under DHEA supplementation, and compares these rates, age-stratified, to miscarriage rates reported in a national USA IVF outcome data base, which involves unselected infertility patients [12]. While study populations at the New York and Toronto centers, thus, involve women with significantly DOR, the national control data reflect only a rather small percentage of women with this primary diagnosis.

DOR patients have in the past resisted prospective randomization. Two registered prospectively randomized, placebo controlled trials, one in new York City and a second in Europe, had to be abandoned for lack of enrollments (Gleicher N and Barad DH, Unpublished data, 2006 and 2007). In the absence of such prospectively controlled studies, the question arose how to establish statistically valid controls for observed miscarriage rates: A control population should involve infertile women under treatment. It also should have maximal size, vary in age distribution (to facilitate age stratification) and be all encompassing (to avoid selection biases). Since here presented DHEA data were generated in North America, a USA-based data base, fulfilling all of these criteria, was chosen [12].

### Definitions

The literature does not offer a unified definition of DOR. Our center now defines all women above age 40 years to suffer from DOR. In women under age 40 the diagnosis is only reached if age-specific ovarian function parameters so indicate [11].

Definitions of clinical pregnancy and of miscarriage follow the reporting requirements of this national data base, defining clinical pregnancy, as confirmed by ultrasound [12].

Since patients at both study centers, as a prerequisite to DHEA supplementation, had to suffer from DOR, their expectation of pregnancy success is very limited [13]. Even considering a higher conception rate in such patients after supplementation with DHEA, [2-4] conceptions will occur in only a small minority of DHEA supplemented cycles. The here reported number of consecutive pregnancies, therefore, represents a range of approximately 450 to 570 initiated DHEA treatment cycles.

### Statistics

Miscarriage rates of DHEA supplemented patients were statistically compared with national IVF outcomes, reported annually under federal mandate by the *Centers for Disease Control and Prevention, U.S. Department of Health and Human Services*. The data utilized as controls for this study reflect 2004 United States IVF statistics [12], report cycle numbers, pregnancy percentages and live birth percentages, stratified for age. These detailed national data allowed calculation of number of clinical pregnancies and number of live births for each age group, since neither is offered in the original data set. We then subtracted live births from pregnancies, to derive number of failed pregnancies (i.e., all failed pregnancies were for purpose of this study considered miscarriages) overall, and in each age category. Counts of pregnancies and miscarriages were then entered into a series of two by two tables, stratified by age, and using the cross tabulation module of SPSS 15.00.

Pregnancy and miscarriage rates at both fertility centers were pooled after confirmation of homogeneity of variance. Common odds ratios of the pooled miscarriage rates among age stratified pregnant patients were compared between the pooled centers and 2004 national rates, utilizing the Mantel-Hänszel common odds ratio (tests for homogeneity of the odds ratio across layers were not significant, meeting assumption for use of this test) and using dichotomous exposure (DHEA versus controls) and dichotomous outcomes (live births versus spontaneous miscarriages), stratified by five age categories.

A secondary statistical analysis of the data was performed, by recalculating for all five investigated age groups (<35, 35-37, 38-40, 40-42 and >42 years) expected miscarriage rates for both patient groups, equalized for size. Both statistical analyses are presented in sequence and were performed using SPSS Windows, standard version 15.0.

### Institutional Review Board

The investigation of DHEA in women with DOR has been repeatedly approved by the center's Institutional Review Board. Since the here reported study only involved the evaluation of (electronic) medical records, and maintained their confidentiality, the here presented study, based on a patient consent signed at time of initial registration, did not require further IRB approval. A confirmatory written statement from the chairman of the IRB is available upon request.

## Results and Discussion

New York reported 40 and Toronto 33 DHEA pregnancies, for a combined DHEA pregnancy experience of 73 pregnancies. Among those pregnancies, New York registered six and Toronto five miscarriages, for clinical miscarriage rates of 15.0% and 15.2%, respectively, and a combined miscarriage rate of 11/73 (15.1%). In comparison, the total 2004 miscarriage rate in the national USA registry was 17.6%. The odds ratio and 95% confidence interval (CI), stratified for age, that a woman would miscarry was, thus, statistically significantly lower after DHEA supplementation [OR 0.49 (0.25 - 0.94; p = 0.04), suggesting a reduction in miscarriage risk of approximately 50 percent (data not shown; Mantel-Hänszel, distributed as Chi-square with one degree of freedom, 4.285; p = 0.038).

When expected miscarriage rates were compared in both patient groups, equalized for number of patients, women after DHEA supplementation demonstrated even more significant reductions in miscarriage rate (p < 0.0001) suggesting an almost 80% reduction in miscarriage risk (data not shown; Mantel-Haenszel, distributed as Chi-square with one degree of freedom, 12.482; p < 0.0001).

**Figure 1:**
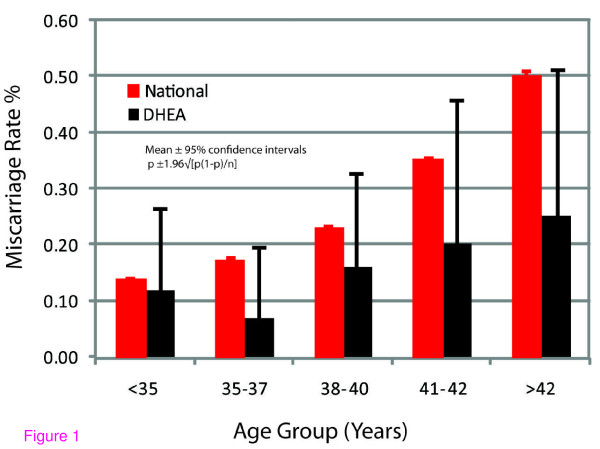
**Comparison of miscarriage rates at all ages between DHEA supplemented infertility patients and 2004 national U.S. IVF outcome data**.

Differences between DHEA treated patients and the national IVF data became even more obvious after age-stratification. Table 1 and Figure 1 summarize age-specific rates in numerical and graphic formats: Miscarriage rates at all ages were lower in DHEA patients than in the 2004 national IVF data. Those differences were, however, only after age 35 years pronounced.

**Table 1:** Age-stratified pregnancy and miscarriage rates

	**Age (years)**				
	**<35**	**35-37**	**38-40**	**41-42**	**>42**
DHEA					
Pregnancies					
NY	10	5	6	10	9
TO	7	10	13	0	3
Miscarriages					
NY	1	0	0	2	3
TO	1	1	3	0	0
					
Misc. Rate (%)					
NY	10.0	0.0	0.0	20.0	33.3
TO	14.3	10.0	23.1	-	0.0
TOTAL	11.8	6.7	15.8	20.0	25.0
(± 95% CI)	(15.0)	(13.0)	(16.0)	(25.0)	(25.0)
					
NATIONAL					
Misc. Rate (%)	14.0	17.1	23.1	36.6	50.1
(± 95% CI)	(1.0)	(1.0)	(1.0)	(2.0)	(5.0)
					
Decrease in Misc. Rate					
with DHEA (%)	-15.7	-60.8	-31.6	-45.3	-50.1

Miscarriage rates after DHEA supplementation, stratified for age, were lower at all ages [OR 0.49 (0.25-0.94; p = 0.04)]. The decrease in miscarriage rate was, however, especially apparent above age 35 years.

NY - Center for Human Reproduction, New York;

TO - Toronto West Fertility Associates, Toronto, Canada

Here reported data, after DHEA supplementation, demonstrate in women with DOR significantly lower miscarriage rates than in a standard IVF control population, - a finding particularly pronounced above age 35 years. This by itself remarkable observation is further enhanced by the well recognized and reported excessive miscarriage risk of women with DOR. Levi et al, for example, reported that women with DOR experience miscarriage rates far in excess of standard IVF patients with normal ovarian reserve: 57.1 percent under age 35; 63.5 percent between ages 35 and 40 and as high as 90 percent above age 40 years [13].

That study patients in the here reported study suffered from DOR is best documented by them receiving DHEA supplementation. Under our center's DHEA protocols, except for women above age 40 years, DHEA supplementation is offered only to women who have failed at least one prior IVF cycle with retrieval of less than four oocytes and, therefore, have been designated resistant to ovarian stimulation. Moreover, younger women receive DHEA supplementation only if they also demonstrate elevated age-specific FSH levels [11]. Finally, DHEA supplementation is voluntary, allowing for the assumption that more severely compromised patients, with poorer past IVF experiences, will more likely choose supplementation.

In contrast, USA IVF outcome data only in a minority represent women with diminished ovarian reserve [12]. As Levi et al. demonstrated [13], control populations, therefore, should demonstrate significantly lower miscarriage rates than our study patients. The finding that women on DHEA supplementation demonstrate in all age groups, but especially above age 35, significantly lower miscarriages than the much more favorable national IVF population is, therefore, noteworthy.

That this difference is less obvious under age 35, only strengthens the validity of the here utilized controls. Indeed, the larger degree of reduction in miscarriage rates in older women should not surprise: Aneuploidy rates increase with age [7-9], and age 35 is generally considered the cut off, when invasive prenatal genetic screening becomes indicated [14]. Assuming a beneficial effect of DHEA on aneuploidy rates, a larger effect after age 35 should, therefore, be expected.

Levi et al. reported in women with diminished ovarian reserve above age 40 an approximately 90% miscarriage rate [13]. Since older women produce fewer embryos, the relative benefits from decreases in aneuploidy rate on number of euploid embryos, transferred into the uterus, will increase with advancing female age [15].

Aneuploidy is, however, even in young women a frequent finding [15,16]. In women with diminished ovarian reserve Levi et al. reported an almost 60 percent miscarriage rate under age 35 years [13]. As women physiologically age, the prevalence of aneuploidy continues to increases, reaching close to 90 percent in the mid- 40s [13,17]. Premature ovarian aging, however, does not prematurely enhance aneuploidy rates, and instead maintains expected age-specific aneuploidy rates [18]. Though demonstrating features of ovarian aging, affected women, therefore, still experience age-appropriate implantation - and pregnancy rates. Because of decreased oocyte and embryo yields, they, however, do demonstrate reduced cumulative pregnancy rates [19,20]. Even though significantly affected by prematurely diminished ovarian reserve, a smaller benefit from DHEA under age 35 in our study population should, therefore, not surprise.

By demonstrating in a very high risk population for spontaneous pregnancy loss a statistical association between DHEA supplementation and decreased miscarriage rates, this study does not prove causation. The study, therefore, does not prove that DHEA decreases miscarriage or aneupoidy rates in human embryos. The here reported data, however, offer enough circumstantial evidence to suggest that DHEA may, indeed, exert both of these effects and, therefore, warrant further investigations. A suggestion of improved euploidy after DHEA supplementation was, after all, also observed in human embryos [6].

Another obvious weakness of the study is the absence of a prospectively established direct control group. Such studies, as our previously noted experience documents, are, however, practically impossible to conduct. Women with severely diminished ovarian reserve often have very limited time to conceive. They, therefore, understandably are resistant to any form of randomization that involves placebo. Because women with DOR, in the absence of DHEA supplementation, rarely conceive [4], expectations for prospectively randomized DHEA studies are not very favorable. Indeed, two attempts at such studies, one in the United States and the other in Europe, had to be abandoned because of insufficient enrollment of women willing to be randomized to placebo (Gleicher N and Barad DH; Unpublished data, 2006 and 2007).

Our center's miscarriage rates in women with DOR, prior to introduction of DHEA supplementation, were, likely, higher than the national rate seen in the here utilized control population [15]. The program's pregnancy rates in these women were then only in low single digits [4]. The gradual introduction of DHEA supplementation between 2004 and 2007 progressively improved pregnancy rates at our center [21]. Increasing pregnancy numbers anecdotally suggested a concomitant decline in miscarriage rates. This observation, in turn, lead to the previously noted investigation of aneuploidy rates in embryos after DHEA supplementation, which, though statistically underpowered, was supportive of a beneficial DHEA effect on ploidy [6].

The New York center's pregnancy and miscarriage data, alone, were, however, not large enough to allow for statistically valid conclusions about factual miscarriage rates. Such conclusions became possible, once the independently collected Toronto data became available, and statistical analysis demonstrated that the two data sets could be unified. At this point the question arose how to control the two centers' miscarriage experiences. A statistical comparison to a large and unselected, national data set appeared appropriate.

While such a comparison cannot replace the gold standard of study design, - the prospectively randomized and placebo controlled study, the here presented data, nevertheless, offer valuable new information. We in this study used carefully vetted statistical methodologies, which are appropriate for the kind of comparisons offered. Moreover, we even performed a second statistical analysis, based on a different statistical model, which suggested an even bigger beneficial statistical effect of DHEA supplementation, increasing the potential benefit from an approximately 50 percent to an approximately 80 percent reduction in miscarriage risk.

Whether the benefit of DHEA supplementation is, indeed, 50 or 80 percent can as of this moment not be ascertained with certainty, but also should not matter. What seems of importance is the observation that DHEA supplementation, at least in women with DOR, who characteristically demonstrate abnormally high miscarriage rates [13], appears to significantly reduce the risk for spontaneous pregnancy loss.

Our here presented data may, at least partially, also help to explain why DHEA supplementation increases egg and embryo quality [1-4], improves pregnancy rates and speeds up time to conception [4]. Egg and embryo quality is, of course, at least partially a reflection of ploidy. More euploid embryos will lead to more pregnancies [22], thus shortening time to conception.

It is important to note that DHEA supplementation, as described, appears safe and results in only minor side effects. Since DHEA is a mild androgen but is converted into testosterone (and estradiol), it should not surprise that observed mild side effects, such as oily skin, mild acne vulgaris and hair loss, are mostly androgenic in nature.

Embryo selection and improving embryo ploidy have been the rationale for attempts at improving pregnancy rates and reducing miscarriage rates via preimplantation genetic screening (PGS) [16,17,22], a concept recently seriously questioned [23,24]. Here presented data suggest that DHEA supplementation may result in more cost effective improvements in ploidy without laboratory intervention.

Though infertile women with normal ovarian reserve experience significantly lower miscarriage rates than DOR patients [13], they still experience higher miscarriage rates than average populations [25]. Here reported miscarriage rates in DHEA treated DOR patients are, therefore, remarkably low and practically identical to those reported for general populations [26,27]. Caution should, nevertheless, be exercised in concluding that observed DHEA effect can automatically be extrapolated to normal, fertile populations, though such a possibility deserves further investigation. If confirmed, one could perceive DHEA as a routine preconception supplement, akin to prenatal vitamins, even in women with no fertility problems.

## Conclusion

Based on the hypothesis that major disturbances in chromosome alignment on the meiotic spindle of oocytes (i.e., congression failure) result from complex interplay of signals, regulating folliculogenesis (increasing the risk of non-disjunction errors), Hodges et al. suggested that it may be possible to develop prophylactic treatments which can reduce the risk of age-related aneuploidy [28]. This study suggests that DHEA may, indeed, be a first such drug.

Should efficacy of DHEA supplementation be proven not only in infertile patients but also in general populations, the potential significance on public health could be considerable and by far exceed the more imminent utilization of DHEA in fertility practice.

## Abbreviations

CI: Confidence interval; CHR: Center for Human Reproduction; DHEA: Dehydroepiandrosterone; DOR: Diminished ovarian reserve; FSH: Follicle stimulating problem; IVF: In vitro fertilization; PGS: Preimplantation genetic screening; PGD: Preimplantation genetic diagnosis.

## Competing interests

NG and DHB are, besides other parties, listed as co-inventors and owners on a patent, which claims therapeutic benefits for DHEA supplementation on ovarian function and pregnancy chances in women with DOR and decreases in miscarriage rates.

## Authors' contributions

NG and DHB contributed equally to the manuscript. Both participated in design of study and data analysis. DHB performed a majority of the statistical analysis, while NG drafted most of the manuscript. ER and SB-M contributed patient data. AW participated in study design and data analysis and all authors read and approved the final manuscript.
